# Network Interactions Explain Sensitivity to Dynamic Faces in the Superior Temporal Sulcus

**DOI:** 10.1093/cercor/bhu083

**Published:** 2014-04-25

**Authors:** Nicholas Furl, Richard N. Henson, Karl J. Friston, Andrew J. Calder

**Affiliations:** 1 MRC Cognition and Brain Sciences Unit, CambridgeCB2 7EF, UK; 2 Wellcome Centre for Imaging Neuroscience, University College London, 12 Queen Square, London WC1N 3BG, UK

**Keywords:** biological motion, dynamic causal modeling, face perception, functional magnetic resonance imaging, superior temporal sulcus

## Abstract

The superior temporal sulcus (STS) in the human and monkey is sensitive to the motion of complex forms such as facial and bodily actions. We used functional magnetic resonance imaging (fMRI) to explore network-level explanations for how the form and motion information in dynamic facial expressions might be combined in the human STS. Ventral occipitotemporal areas selective for facial form were localized in occipital and fusiform face areas (OFA and FFA), and motion sensitivity was localized in the more dorsal temporal area V5. We then tested various connectivity models that modeled communication between the ventral form and dorsal motion pathways. We show that facial form information modulated transmission of motion information from V5 to the STS, and that this face-selective modulation likely originated in OFA. This finding shows that form-selective motion sensitivity in the STS can be explained in terms of modulation of gain control on information flow in the motion pathway, and provides a substantial constraint for theories of the perception of faces and biological motion.

## Introduction

Humans and other animals effortlessly recognize facial identities and actions such as emotional expressions even when faces continuously move. Brain representations of dynamic faces may be manifested as greater responses in the superior temporal sulcus (STS) to facial motion than motion of nonface objects (Pitcher et al. 2011), suggesting localized representations that combine information about motion and facial form. This finding relates to a considerable literature on “biological motion,” which studies how the complex forms of bodily actions are perceived from only the motion of light points fixed to limb joints, with form-related texture cues removed (Johansson 1973). Perception of such stimuli has been repeatedly associated with the human posterior STS (Vaina et al. 2001; Vaina and Gross 2004; Giese and Poggio 2003; Hein and Knight 2008; Jastorff and Orban 2009) with similar results observed in potentially corresponding areas of the macaque STS (Oram and Perrett 1994; Jastorff et al. 2012). The STS has been described as integrating form and motion information (Vaina et al. 2001; Giese and Poggio 2003), containing neurons that code for conjunctions of certain forms and movements (Oram and Perrett 1996). Nevertheless, the mechanisms by which STS neurons come to be sensitive to the motion of some forms, but not others, remains a matter of speculation (Giese and Poggio 2003).

We propose that network interactions can provide a mechanistic explanation for STS sensitivity to motion that is selective to certain forms, in this case, faces. Specifically, STS responses to dynamic faces could result from communicative interactions between pathways sensitive to motion and facial form. Such interactions can occur when one pathway modulates or “gates” the ability of the other pathway to transmit information to the STS. Using functional magnetic resonance imaging (fMRI), we localized face-selective motion sensitivity in the STS of the human and then used causal connectivity analyses to model how these STS responses are influenced by areas sensitive to motion and areas selective to facial form. We localized ventral occipital and fusiform face areas (OFA and FFA) (Kanwisher et al. 1997), which selectively respond to facial form versus other objects (Calder and Young 2005; Calder 2011). We also localized motion sensitivity to faces and nonfaces in the more dorsal temporal hMT+/V5 complex (hereafter, V5). Together, these areas provide ventral and dorsal pathways to the STS. The ventral pathway transmits facial form information, via OFA and FFA, and the dorsal pathway transmits motion information, via V5. We then compared combinations of bilinear and nonlinear dynamic causal models (Friston et al. 2003) to identify connectivity models that optimally explain how interactions between these form and motion pathways could generate STS responses to dynamic faces. We found that information about facial form, most likely originating in the OFA, gates the transmission of information about motion from V5 to the STS. Thus, integrated facial form and motion information in the STS can arise due to network interactions, where form and motion pathways play distinct roles.

## Materials and Methods

### Participants

fMRI data were collected from 18 healthy, right-handed participants (over 18 years, 13 females) with normal or corrected-to-normal vision. Experimental procedures were approved by the Cambridge Psychology Research Ethics Committee.

### Imaging Acquisition

A 3T Siemens Tim Trio MRI scanner with a 32-channel head coil was used for data acquisition. We collected a structural *T*_1_-weighted MPRAGE image (1-mm isotropic voxels). Functional data consisted of whole-brain *T*_2_*-weighted echo-planar imaging volumes with 32 oblique axial slices that were 3.5 mm thick, in-plane 64 × 64 matrix with resolution of 3 × 3 mm, TR 2 s, TE 30 ms, flip angle 78°. We discarded the first 5 “dummy” volumes to ensure magnetic equilibration.

### Experimental Design

The experiment used a block design with 2 runs (229 scans per run), which were collected as the localizer for another experiment (Furl, Henson, et al. 2013). Note that the dynamic causal modeling (DCM) analyses reported in Furl, Henson et al. (2013) used independent data (from separate runs using different stimuli) to address a different phenomenon than considered here. All blocks were 11 s, comprised 8 1375 ms presentations of grayscale stimuli and were followed by a 1-s interblock fixation interval. Participants fixated on a gray dot in the center of the display, overlaying the image. On a random one-third of stimulus presentations, this dot turned red and they pressed a key. Participants viewed 6 types of blocks, each presented 6 times. Dynamic face blocks contained dynamic facial expressions taken from the Amsterdam Dynamic Facial Expression Set (ADFES) (van der Schalk et al. 2011). Four male and four female identities changed among neutral and either disgust, fearful, happy, or sad expressions. Identities and expressions appeared in a pseudo-random order, with each of the 4 expressions appearing twice in each dynamic face block. Dynamic object blocks included 8 dynamic objects (Fox et al. 2009). For comparison, we also included dynamic and static patterns. We used a conventional low-level motion localizer, commonly used to localize and study motion sensitive areas hMT+/V5 and KO (van Oostende et al. 1997). This ensured that our results are directly comparable with previous studies of low-level motion sensitivity and verifies that the V5 voxels we identify using faces are a subset of hMT+/V5 voxels, as conventionally defined. These dynamic pattern blocks consisted of random-dot pattern videos with motion-defined oriented gratings. The stimuli depicted 50% randomly luminous pixels, which could move at one frame per second horizontally, vertically, or diagonally left or right. Oriented gratings were defined by moving the dots within 4 strips of pixels in the opposite direction to the rest of the display, but at the same rate (van Oostende et al. 1997). The remaining 3 block types—static face, object, and pattern blocks—consisted of the final frames of the corresponding dynamic blocks.

### Preprocessing and Analysis

We performed preprocessing and analysis using SPM8, DCM10 (Wellcome Trust Centre for Neuroimaging, London http://www.fil.ion.ucl.ac.uk/spm/) and MATLAB (The MathWorks, Natick, MA, USA). Data were motion and slice-time corrected, spatially normalized to an EPI template in MNI space, smoothed to 8-mm full-width half-maximum and analyzed using the general linear model. At the first (within-participant) level, general linear models used proportionately scaled data, an AR(1) autocorrelation model, a high-pass filter of 128 s and regressors constructed by convolving the onset times and durations for the different experimental blocks with a canonical hemodynamic response function.

At the first level, we localized face-selective regions of interest (ROIs) in the right OFA and FFA by contrasting the average response to dynamic and static faces versus the average response to dynamic and static objects and random-dot patterns. We also identified an ROI showing motion sensitivity to faces in the vicinity of area hMT+/V5 (V5) by contrasting dynamic versus static faces. We further localized an area in the STS by computing the interaction effect in which motion sensitivity was larger for faces than for nonfaces using the contrast (dynamic faces > static faces) > (dynamic objects/patterns > static objects/patterns). Lastly, we contrasted faces, objects, and patterns versus fixation to localize the peak visual response, which was located in right Brodmann area 18 (BA18). For BA 18, we located the peak response to faces, objects and patterns in the whole sample of 18 participants (MNI: 16 −90 −4) and then identified subject-specific peaks within 8 mm of the group peak. Eleven of the 18 participants evidenced significant responses (at *P* < 0.01 uncorrected) in all 5 ROIs in the right hemisphere and further analyses focused on these ROIs—given the right hemispheric dominance in face perception (Kanwisher et al. 1997). Note that our selection of ROIs for subsequent DCM analyses is slightly more conservative than standard approaches. This is because we chose subject-specific maxima that were within a specified distance of peaks in an orthogonal contrast (at the group level) (cf., Friston 1997). In other words, they were selected using orthogonal (independent) criteria, rendering a correction for multiple comparisons redundant.

For connectivity analysis, we employed DCM (Friston et al. 2003) to test hypotheses about connectivity mechanisms that potentially could give rise to the selective facial motion sensitivity that we observed in the STS. DCM models ROI time series data by estimating coupling: The extent to which neural activity (hidden variables) in each brain area influences dynamics in connected brain areas. DCM parameters include exogenous inputs, endogenous connections, bilinear, and nonlinear modulatory connections. Exogenous inputs are estimates of the perturbation of the neuronal states by stimulus presentations; in this case, faces, objects, and random-dot patterns. Endogenous connections reflect directed coupling among areas, averaged over experimental conditions. Connections with bilinear modulation show changes in coupling induced by an experimental factor. Connections in our models could be bilinearly modulated by the presence of motion or by facial form. Nonlinear modulations reflect changes in coupling induced by another ROI. Note that nonlinear modulations can be used to explain bilinear effects. For example, bilinear modulation of faces versus nonfaces might arise on a connection because it is nonlinearly modulated by a face-selective area. We used nonlinear parameters to examine how areas in one pathway (e.g., facial form pathway) affect information flow in the other pathway (e.g., motion pathway). With DCM, we varied the presence or absence of endogenous, bilinear, or nonlinear parameters and performed Bayesian model comparisons with identify the optimal model architecture. We first compared a bilinear model space, where we identified the model that best explained how bilinear influences of motion and facial form explain STS responses. We then performed a second model comparison, using nonlinear models, to identify the brain areas whose activity could optimally account for the motion and facial form modulation we observed in the optimal bilinear model (see below).

Before model comparison, we formulated a “base model” that accounted for: 1) the fact that the entire network is driven by face and nonface stimuli (objects and patterns), and 2) that OFA and FFA respond preferentially to faces, while V5 responds preferentially to motion. We drove the network by face, object and pattern stimulation by including an input area (BA18) that responded to these three stimuli and is in a position to propagate neuronal signals throughout the network. This BA18 area corresponds to, low-level visual cortex, which is known to respond to visual stimuli generally and to feedforward its responses to higher visual areas. Consistent with this role for BA18, we accounted for face selectivity by adding (bilinear) modulation by faces to the connection from BA18 to OFA. Similarly, we accounted for motion sensitivity by adding modulation by motion to the connection from BA18 to V5. Model comparison then proceeded by varying other properties of this base model.

We compared individual models (Table 1) and model families (Table 2) using their relative log-evidences and posterior probabilities—assuming all participants used the same connectivity architecture (Penny et al. 2004, 2010; Stephan et al. 2010). The main focus of our model comparisons was to determine whether motion sensitivity that is selective to facial form in the STS could be explained by network interactions between motion and facial form pathways. We considered two alternative mechanisms for this interaction. First, the connection between a face-selective area (OFA and/or FFA) and the STS could be modulated by motion. Second, the connection between a motion-sensitive area (V5) and STS could be modulated by facial form. We first cast these hypotheses in the form of bilinear models, and performed a model comparison using 16 models (cells in Table 1). These 16 models were divided into four model families, corresponding to the mechanisms that could produce the form by motion interaction in STS (see columns in Table 1). These four families tested 1) face modulation of the motion pathway to STS from V5, 2) motion modulation of the face pathway to STS from OFA, 3) motion modulation of the face pathway to STS from OFA, and 4) motion modulation of both face pathways from OFA and FFA. These 4 families were crossed with 2 other variants of model, which tested incidental hypotheses, as shown in the rows of Table 1. First, the bilinear models could be either “full connectivity,” with all possible endogenous connections, or the connectivity could be sparse. The sparse models were motivated by a previous study of magnetoencephalographic induced responses that showed no endogenous connectivity between FFA and the STS and only feedforward connections (Furl, Coppola, et al. 2014). Second, the bilinear models either possessed modulation by faces on only the connection from BA18 to OFA (“OFA only” rows in Table 1) or possessed modulation on the connections from BA18 to both OFA and FFA (“OFA/FFA” rows in Table 1).

**Table 1:** Bilinear model evidences and posterior probabilities

	Faces modulate V5 to STS	Motion modulates OFA to STS	Motion modulates FFA to STS	Motion modulates OFA/FFA to STS
Full				
OFA only	**285.20** **(1)**^a^	231.47 (0)	222.64 (0)	87.92 (0)
OFA/FFA	74.15 (0)	81.65 (0)	92.11 (0)	55.79 (0)
Sparse				
OFA only	12.71 (0)	9.19 (0)	9.49 (0)	0 (0)
OFA/FFA	12.62 (0)	9.13 (0)	9.43 (0)	0.09 (0)

^a^We compared 16 bilinear DCMs on the basis of their model evidences (with posterior probabilities shown in parentheses). The highest evidence model is shown in bold.

**Table 2:** Bilinear family model evidences and posterior probabilities^a^

Faces modulate V5 to STS	2.89 (1)
Motion modulates OFA to STS	0 (0)
Motion modulates FFA to STS	0 (0)
Motion modulates OFA/FFA to STS	0 (0)
Full	2.89 (1)
Sparse	0 (0)
OFA only	2.89 (1)
OFA/FFA	0 (0)

^a^We compared evidences (with posterior probabilities shown in parentheses) aggregated over “families” of bilinear DCMs that shared specific features of interest. The first 4 rows compare 4 families that could each differently explain the face-specific motion sensitivity in the STS. The fifth and sixth rows compare families with full versus sparse endogenous connectivity. The seventh and eighth rows compare a family using modulation of faces on the connection from BA18 to OFA versus a family using modulation on connections from BA18 to both OFA and FFA.

Our bilinear model comparison revealed that facial form information modulated the connection from V5 to STS (see Results for more information). However, this result does not identify the mechanism that causes this modulation. To do this, we used nonlinear models in which face-selective areas can directly influence the connection from V5 to STS. Here, we could test whether the face-selective responses in OFA, FFA, or both influenced the motion information propagating to STS from V5. Nonlinear influences from these face-selective areas could account for the bilinear modulation of faces that we observed. Note that, in principle, it would be preferable to test all our hypotheses in one nonlinear model space. In this case, we would have compared nonlinear models where face-selective areas influence the connection from V5 to STS against nonlinear models where the motion-sensitive area V5 influences connections from the face-selective areas. However, the multiplicative nature of nonlinear terms (Stephan et al. 2008) results in mathematically symmetrical nonlinear DCMs, preventing this model comparison in practice. We therefore first tested bilinear models which showed that faces modulated the connection from V5 to STS and then we tested nonlinear models to identify a possible face-selective area responsible for the bilinear modulation of faces.

## Results

### ROI Specification

**Figure 1.**
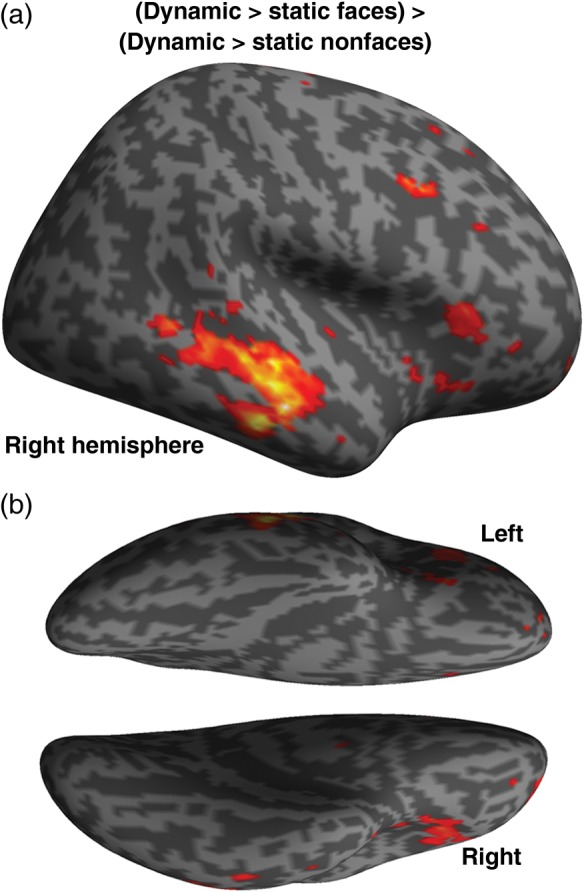
Group-level whole-brain analysis. (*a*) Results of contrast (dynamic faces > static faces) > (dynamic nonfaces > static nonfaces). (*b*) Voxels showing significant effects at *P* < 0.005 (uncorrected) are projected on an inflated cortical surface of the right hemisphere in MNI space. STS, superior temporal sulcus.

We located ROIs in individual participants. We used the contrast of faces, objects and patterns versus fixation to identify BA18; the contrast of dynamic and static faces versus dynamic and static objects and patterns to identify the conventional face-selective areas OFA and FFA; the contrast of dynamic versus static faces to identify the motion-sensitive area V5; and the contrast (dynamic faces > static faces) > (dynamic objects/patterns > static objects/patterns) to identify face-specific motion sensitivity in the STS. For display purposes, Figure 1 illustrates the results of this contrast in the STS at the group level, using the 11 participants who showed every ROI (peak voxel MNI: 56 −24 −8). This STS area was observed at *P* < 0.005 uncorrected where it also met the *P* < 0.0001 threshold for familywise error correction at the cluster level (Brett et al. 2003).

### Group-Level ROI Analyses

**Figure 2.**
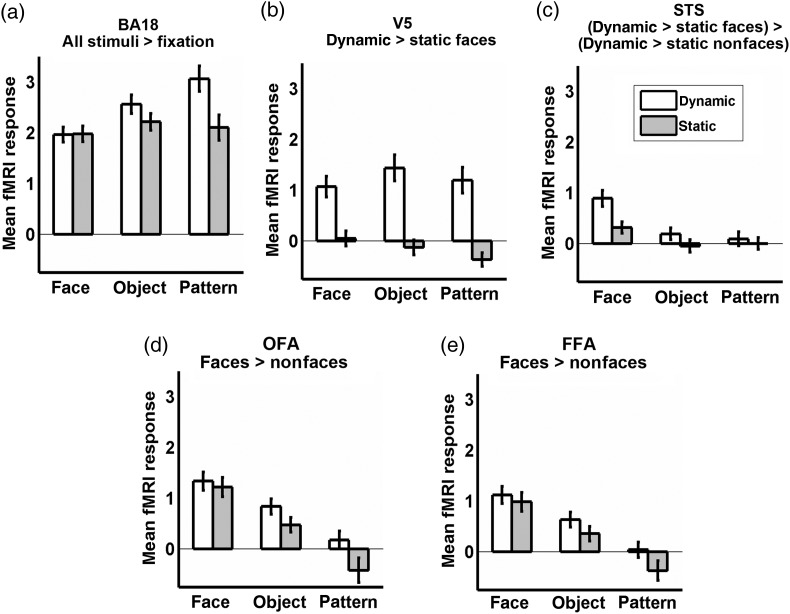
Group-level region-of-interest (ROI) analysis. (*a*) Mean responses in Brodmann area 18 (BA18) to faces, objects, and random-dot patterns; (*b*) mean responses in V5; (*c*) mean responses in the superior temporal sulcus (STS); (*d*) mean responses in the occipital face area (OFA); (*e*) mean responses in the fusiform face area (FFA). Graph titles describe contrast used to define ROI.

Figure 2 shows the response patterns in our ROIs at the group level using ANOVAs with motion (dynamic or static) and category (face, object, or pattern) as factors, followed by post hoc tests (Tukey honest significant difference corrected *P* < 0.05). Some of the ANOVA effects duplicate the contrasts used to define the ROIs including the main effect of category in face-selective ROIs and the mean effect of motion in motion-sensitive ROIs. We include these tests here for completeness and to illustrate the quantitative patterns of means within the voxels identified in the ROIs However, our main conclusions from the ROI analyses are drawn from orthogonal ANOVA effects to preclude biased inferences. These include effects of motion in face-selective ROIs and effects of category in motion-sensitive ROIs.

BA18 (Fig. 2*a*) showed robust responses in every condition, with enhanced responses to dynamic patterns, resulting in a motion × category interaction (*F*_1,50_ = 9.30, *P* < 0.001) and a significant pairwise difference between dynamic and static patterns (there were no other significant pairwise effects). V5 (Fig. 2*b*) showed robust responses to all dynamic stimuli, with no positive responses to any static stimulus, and significant differences between dynamic versus static versions of all 3 categories of stimuli, resulting in our hypothesized main effect of motion *F*_1,50_ = 304.65, *P* < 0.001. Because motion sensitivity was numerically smaller for faces than for objects and patterns, there was a motion × category interaction (*F*_1,50_ = 9.52, *P* = 0.009). The STS also showed a motion × category interaction (*F*_1,50_ = 18.72, *P* < 0.001), but because of a different response pattern than for V5 and BA18. In the STS, pairwise tests showed significant motion sensitivity only for faces, but not for objects or random-dot patterns. Neither ventral area showed any motion × category interaction (OFA: *P* = 0.077; FFA: *P* = 0.264), although we detected main effects of motion (OFA: *F*_1,50_ = 17.73, *P* < 0.001; FFA: *F*_1,50_ = 16.51, *P* < 0.001) in addition to the main effect of category (OFA: *F*_1,50_ = 91.06, *P* < 0.001; FFA: *F*_1,50_ = 108.79, *P* < 0.001). Closer inspection using pairwise tests showed that the main effect for OFA was driven by motion sensitivity for patterns but no significant motion sensitivity for faces or objects. For the FFA, no category showed significant motion sensitivity when tested alone. In summary, only the STS showed motion sensitivity that was selective for faces. V5 showed motion sensitivity to faces as well as objects and patterns, while BA18, the OFA and FFA showed no evidence for motion sensitivity to faces.

### Connectivity Models

**Figure 3.**
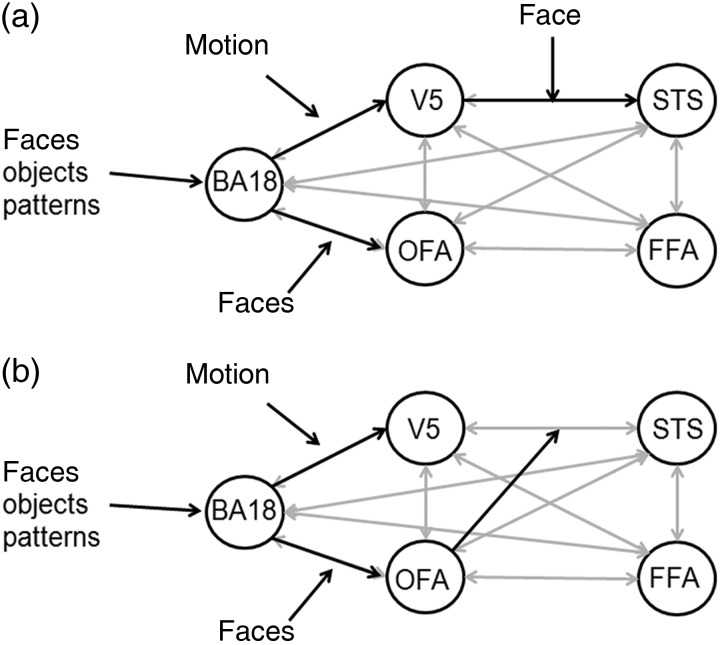
Optimal dynamic causal models (*a*) the optimal bilinear model generates motion sensitivity that is selective to facial form in the superior temporal sulcus (STS) when faces modulate connections from the motion-sensitive V5 to STS. Bilinear modulations indicated by black arrows, endogenous connections indicated in light gray. The optimal model had full endogenous connectivity. (*b*) The optimal nonlinear model shows that the face-selective occipital face area (OFA) is the most likely origin of face modulation on the connections from V5 to STS. Bilinear and nonlinear modulations indicated by black arrows, endogenous connections indicated in light gray. FFA, fusiform face area, BA18, Brodmann area 18.

Our ROI analysis confirmed the presence of dorsal temporal motion sensitivity in V5, facial motion sensitivity in the STS, and ventral temporal face selectivity in the OFA and FFA. We used connectivity modeling to test how interactions between the dorsal motion-sensitive and the ventral face-selective pathways could give rise to motion sensitivity that is selective to faces in the STS. We first compared bilinear models to test whether STS responses might be explained by a network, either in which faces modulate dorsal motion-sensitive pathway connections from V5 to STS (Fig. 3), or in which motion modulates the ventral face-selective pathway connections from the OFA and/or FFA to the STS. This space of bilinear models further explored as secondary hypotheses whether (*a*) endogenous connectivity is full or sparse and (*b*) face selectivity in the ventral pathway arises from modulation by faces on only forward connections to the OFA, or if forward connections to the FFA are modulated by faces as well (OFA only and OFA/FFA rows in Table 1). Of the 16 models we tested, we found a high posterior probability (near 1.0) favoring a model where faces modulate the dorsal motion-sensitive connections from V5 to the STS. For our secondary hypotheses, we found (*a*) full (rather than sparse) endogenous connectivity and (*b*) face modulation on connections from BA18 to the OFA only (and not also to the FFA). These properties of the optimal model were confirmed using model family comparisons (Table 2).

Having established that faces modulate the dorsal motion-sensitive connection from V5 to the STS, we assumed that this face modulation arose from the activity in a face-selective area in the ventral pathway. We therefore used 3 additional nonlinear models to test whether face modulation on the dorsal motion-sensitive connections from V5 to the STS was more likely to arise from face-selective responses in OFA or FFA or both. We found a near-perfect posterior probability favoring the model where the OFA, but not the FFA (nor both), modulates the connection from V5 to the STS.

## Discussion

We show that motion sensitivity to facial form in the STS was best explained by a DCM where transmission of motion information from V5 to the STS is gated or modulated by information about facial form. Face-selective responses in the OFA most likely implemented this gating. This model provides a network-based account for the emergence of face-selective motion sensitivity in the STS and, perhaps, could also explain the integration of motion and form information when viewing biological motion.

Responses to biological motion constitute a type of form-selective motion sensitivity, in the sense that they respond only to conjunctions of motion with specific forms. Consequently, studies in this area often characterize perception of biological motion as resulting from a mixture of contributions of form and motion representations (Thompson and Baccus 2012), which may be transmitted by separate occipitotemporal pathways (Giese and Poggio 2003) and may converge on the STS, where the form and motion information is combined (Oram and Perrett 1996; Vaina et al. 2001; Thompson et al. 2005; Lange and Lappe 2006). Not surprisingly, the dominant theoretical frameworks from the face perception literature are similarly structured, with distinct pathways representing facial form and movements. Low-level facial feature information might be processed in the OFA and then fed-forward into dorsal and ventral pathways (Haxby et al. 2000). Information about static form or invariant facial features is considered to be represented in ventral areas like the OFA and FFA (O'Toole et al. 2002; Calder and Young 2005; Calder 2011; Haxby and Gobbini 2011), which are selective for facial form (Kanwisher et al. 1997). More dorsal areas, such as the STS (Haxby et al. 2000; Haxby and Gobbini 2011) and V5 (O'Toole et al. 2002), however, are more sensitive to facial motion than OFA and FFA (Schultz and Pilz 2009; Trautmann et al. 2009; Pitcher et al. 2011; Foley et al. 2012; Grosbras et al. 2012; Schultz et al. 2013). These dorsal areas may employ motion-based representations to recognize the changeable aspects of faces (Haxby and Gobbini 2011; Foley et al. 2012). While our results suggest that the STS is driven by facial motion information, they further show that STS responses are not dependent on a single, motion-based pathway, but instead are the result of nonlinear interactions between motion and form pathways.

A previous study using connectivity analyses (Foley et al. 2012) showed that responses in inferior occipital gyrus and STS were more correlated for dynamic than for static faces. Indeed, a model like this could plausibly explain the form by motion interaction that we observed in the STS. In this case, the STS would receive signals from OFA that are already form-dependent (because OFA is face-selective) and the addition of motion modulation on the OFA to STS connection would introduce an interaction of form and motion in the STS. However, our bilinear model space tested a family of models with this property (Table 1, column 2, Table 2, row 2) and it was suboptimal, compared with another means of introducing a form by motion interaction in STS. The more likely model family showed that facial form modulated the motion-sensitive responses conveyed to STS from V5 (Table 1, column 1, Table 2, row 1). We then showed that this facial form modulation could occur when OFA activity (which is selective to facial form) nonlinearly modulates the flow of motion information from V5 to STS. In other words, the OFA acted as a modulatory gain control on the “driving signal” in the motion pathway, rather than simply conveying the motion information itself (Foley et al. 2012). These nonlinear interactions also go beyond previous work because they predict hypothetical neural mechanisms (Stephan et al. 2008), where a neural population in the OFA might introduce short-term synaptic plasticity in its target (the STS) by altering its receptivity to other neural populations that drive it (V5). Our results therefore provide neural-level hypotheses to be explored in the nonhuman primate, which has well-characterized visual areas sensitive to faces (Tsao et al. 2006) as well as motion (Dubner and Zeki 1971; Desimone and Ungerleider 1986; Nelissen et al. 2006), including biological motion (Oram and Perrett 1994, 1996; Nelissen et al. 2011).

Our study focused on explaining STS motion-sensitive responses to faces versus objects. However, some areas in the STS are well known to be generally sensitive to biological forms. Our results suggest a mechanism that might generalize to integration of motion and form in cases of biological motion, although this requires confirmation using speech movements, grasping actions, or point-light displays. We can claim that our STS area is not involved simply in representing low-level motion or motion-defined shape features, because it did not show sensitivity to random-dot patterns with motion-defined contours. We can also claim that our STS area did not show sensitivity to motion that depicts complex forms, as it was not sensitive to object motion (Beauchamp et al. 2002, 2003; Pitcher et al. 2011). However, we do not know how sensitive our STS area is to nonface body movements. There is evidence that different areas in the STS show sensitivity to specific body parts (Wheaton et al. 2004; Thompson et al. 2007; Grosbras et al. 2012). However, motion sensitivity to different stimuli may overlap as well. The posterior STS responds in common to a variety of different types of movements when they are compared with scrambled movements without form cues (Santi et al. 2003; Thompson et al. 2007; Grosbras et al. 2012). And similar areas in the posterior STS are associated with point-light body actions as well as faces (Hein and Knight 2008). The interaction of facial form and motion we observed, however, showed its peak effect in a more anterior area of STS than that commonly observed for point-light displays of bodily actions. Thus, any overlap between the STS area we observed and motion sensitivity to other types of complex stimuli such as bodies still needs to be established.

Our results suggest that access of facial motion to the STS is dependent on an occipital area that is selective to facial form, the OFA. It remains to be seen whether other form-selective areas perform similar gating on motion information in other stimulus domains. For example, the extrastriate or fusiform body areas might gate connections between V5 and the STS during body perception. Hein and Knight (2008) hypothesized that STS responses to actions associated with theory of mind inferences or audiovisual speech movements might be dependent, respectively, on responses in medial and inferior prefrontal areas. It remains unclear whether these areas might have a driving (like V5) or a gating/modulatory (like the OFA) relationship with STS responses. Inferior frontal cortex, in particular, has been implicated in perception of facial and other types of biological motion (Saygin et al. 2004; Wheaton et al. 2004; Casile et al. 2010; Furl et al. 2010; van Kemenade et al. 2012). Indeed, inferior frontal involvement has been characterized as a top-down process involving motor representations coded by mirror neuron responses (Caggiano et al. 2011; Kilner 2011; Nelissen et al. 2011). We did not observe reliable inferior frontal responses in our individual participants useful for modeling using our current data. However, connectivity analyses like DCM may provide a powerful technique for measuring top-down influences on STS responses to dynamic visual stimuli.

In summary, we present a connectivity model of fMRI data that explains, in terms of network dynamics, the origin of motion sensitivity that is selective to facial form in the STS. We demonstrate how responses in the STS can depend on interactions between information flow in a dorsal motion-sensitive pathway and a ventral facial form-selective pathway. The presence of information about facial form enhanced the ability of the motion-sensitive area V5 to influence responses in the STS. This gain control modulation likely originated in the OFA. Our model of network interactions provides a plausible mechanistic explanation for how form and motion information are integrated when viewing biological motion. This new perspective on network-level causes of brain responses to dynamic stimuli opens several future research avenues.

## Funding

This work was supported by funding from the United Kingdom Economic and Social Research Council (RES-062-23-2925) to N.F. A.J.C. (MC_US_A060_5PQ50) and R.N.H. (MC_US_A060_0046) are supported by the Medical Research Council. Funding to pay the Open Access publication charges for this article was provided by the United Kingdom Economic and Social Research Council RES-062-23-2925.
